# Supplementary material for: Deriving disease modules from the compressed transcriptional space embedded in a deep autoencoder
Source: Nat Commun. 2020 Feb 12;11:856. doi: 10.1038/s41467-020-14666-6 (PMC7016183; doi:10.1038/s41467-020-14666-6)
Supplement: Supplementary file 1 — Supplementary Information [file 41467_2020_14666_MOESM1_ESM.pdf]

Deriving Disease Modules from the Compressed Transcriptional Space Embedded in a Deep Auto-encoder

Dwivedi et al.

Supplementary Table 1 | Disease association enrichment scores, median value, from Fisher's exact test using control and disease related gene expression samples

Disease	Cell-type	P-value for control	OR for control	Count for control	P-value for disease	OR for disease	Count for disease
Multiple sclerosis	Cerebrospinal fluid (CSF)	8.64×10^{-3}	1.74	22	1.12×10^{-5}	2.37	30
Obesity	Adipose tissue	1.00×10^{-7}	2.40	43	1.28×10^{-8}	2.52	45
Ulcerative colitis	Colonic mucosa	3.90×10^{-4}	2.14	24	6.16×10^{-5}	2.32	26
Crohn's disease	Colonic mucosa	1.02×10^{-2}	1.71	22	2.33×10^{-4}	2.10	27
Asthma	Lymphoid	6.89×10^{-3}	1.59	32	2.89×10^{-4}	1.83	37
Colon carcionoma	Colorectal	1.75×10^{-1}	1.48	8	4.49×10^{-2}	1.84	10
Colorectal carcionoma	Colorectal	1.36×10^{-4}	2.05	31	3.82×10^{-6}	2.31	35
Non-Small Cell Lung Carcinoma	Lung	1.01×10^{-3}	2.21	19	5.71×10^{-3}	1.98	17

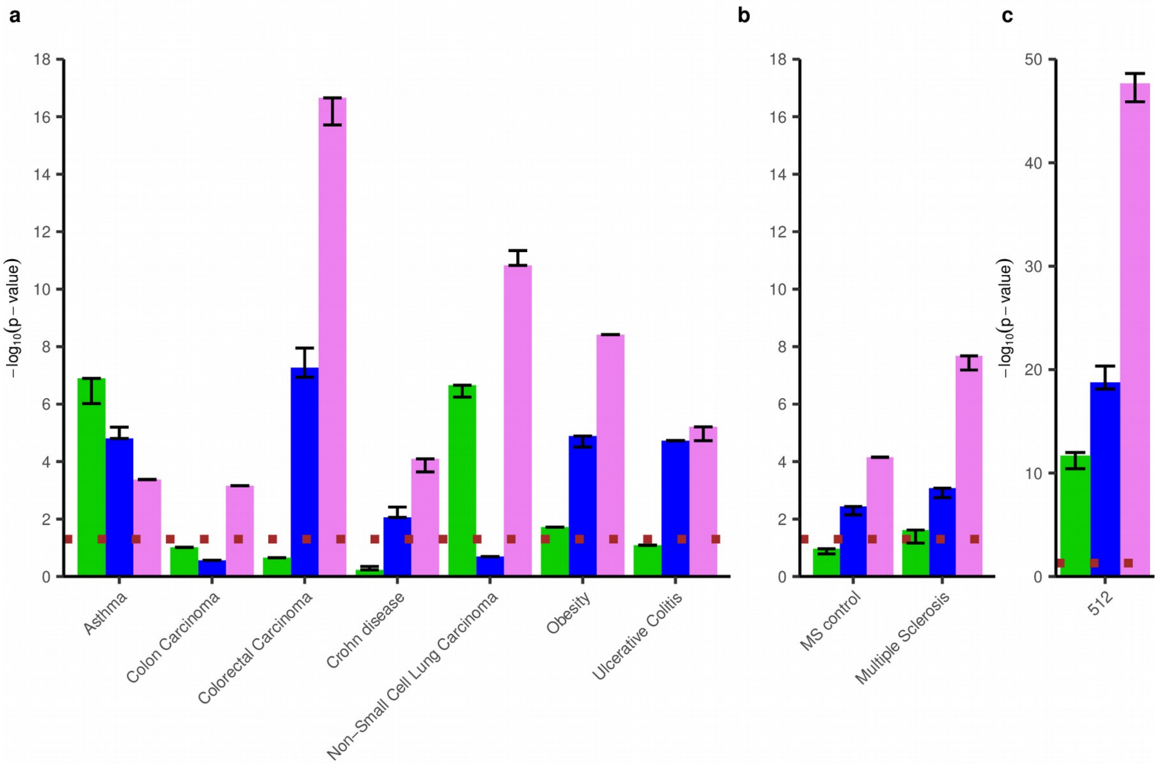

Supplementary Figure 1: Disease ontology[1] gene set enrichment of predicted gene by compressed representations of auto-encoder (AE) trained on the microarray data. Enrichment score (-log10(P)) resulting from hyper-geometric test between disease gene overlap of the predicted genes by the deep neural network derived by first (green), second (blue), and third (violet) hidden layers of the deep auto-encoder (deepAE). The dotted (brown) line corresponds to the p-value, cut-off 0.05 in the independent validation set in the case of control vs. MS. Panel (c) demonstrates the Fisher's combined p-value across all eight diseases predicted by a 3-layer deep auto-encoder.

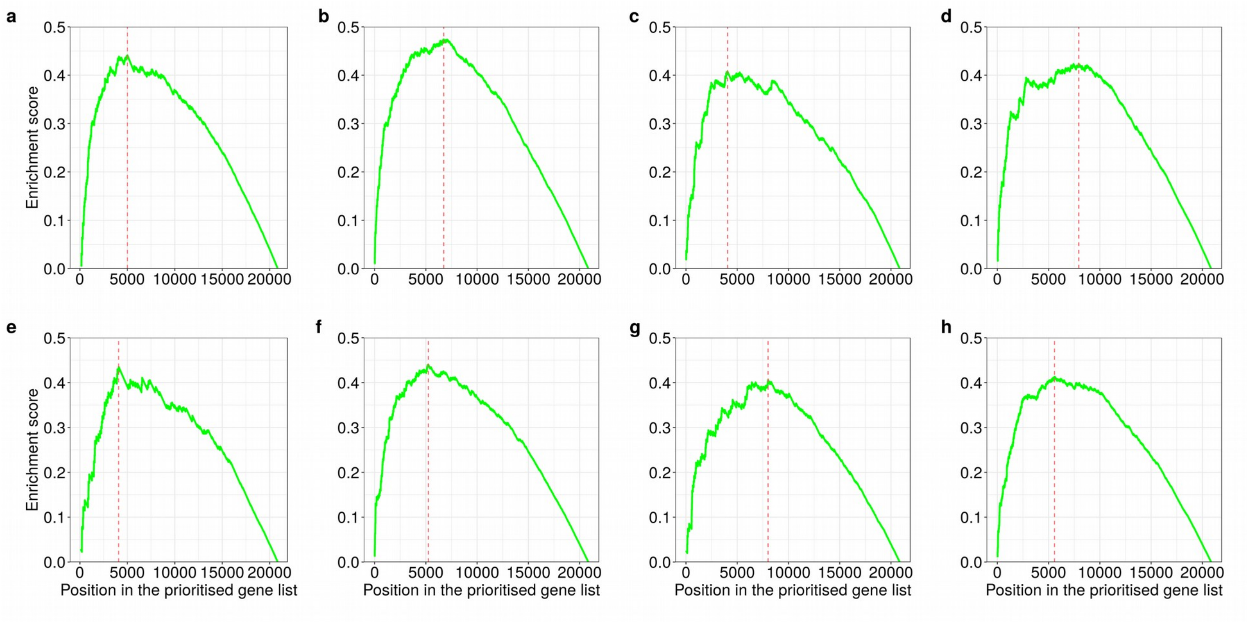

Supplementary Figure 2: GWAS associated disease gene set enrichment analysis (GSEA) score of predicted gene by third layer compressed representations of the deep auto-encoder (deepAE) trained on the microarray data. The Benjamini-Hochberg (BH) adjusted p-value, computed using clusterProfiler of R package[2], for Multiple sclerosis (a), Obesity (b), Crohn's disease (c), Ulcerative colitis (d), Colon carcinoma (e), Colorectal carcinoma (f), Non-small-cell lung carcinoma (g) and Asthma (h) are 5.4×10^{-5}, 1.0×10^{-7}, 3.8×10^{-3}, 1.3×10^{-3}, 1.0×10^{-2}, 5.0×10^{-6}, 1.1×10^{-2} and 1.8×10^{-5} respectively.

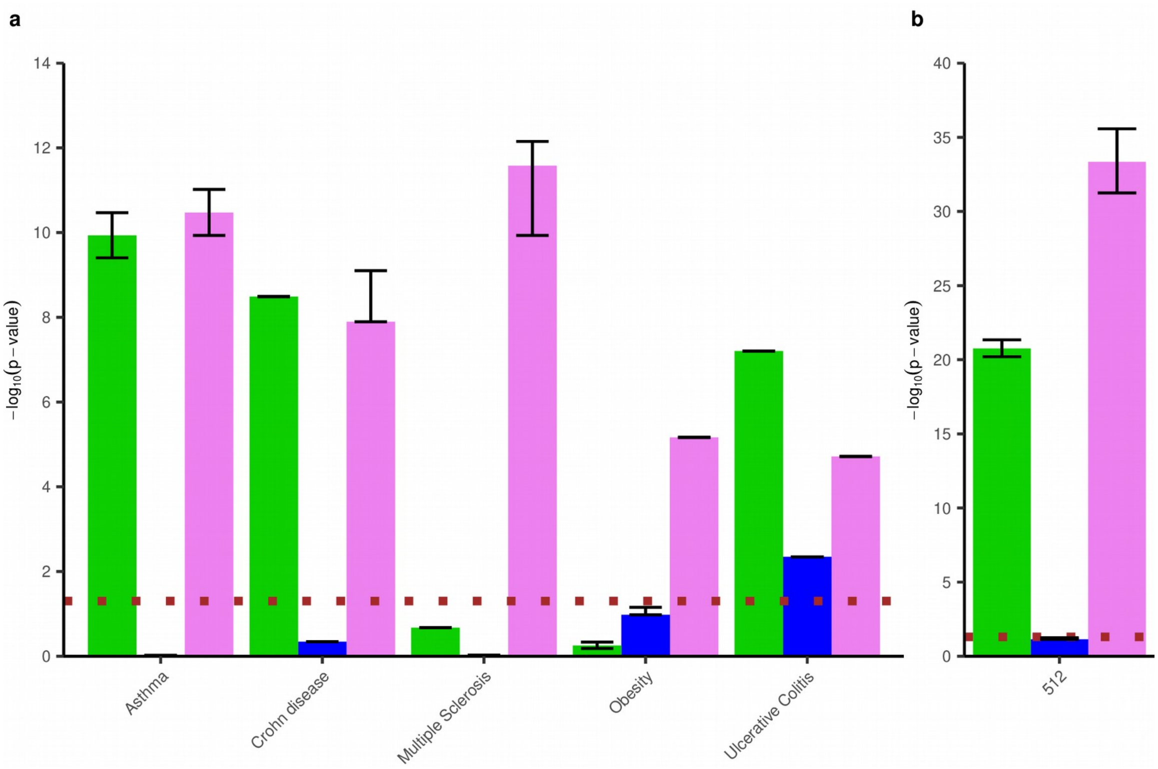

Supplementary Figure 3: Disease ontology[1] gene set enrichment of predicted genes by compressed representations auto-encoder (AE) trained on the RNAseq data. Enrichment score (-log10(P)) resulting from hyper-geometric test between disease gene overlap of the predicted genes by the deep neural network derived by first (green), second (blue), and third (violet) hidden layers of the deep auto-encoder (deepAE). The dotted (brown) line corresponds to the p-value, cut-off 0.05 in the independent validation set in the case of control vs. MS. Panel (C) demonstrates the Fisher's combined p-value across all eight diseases predicted by a 3-layer deep auto-encoder.

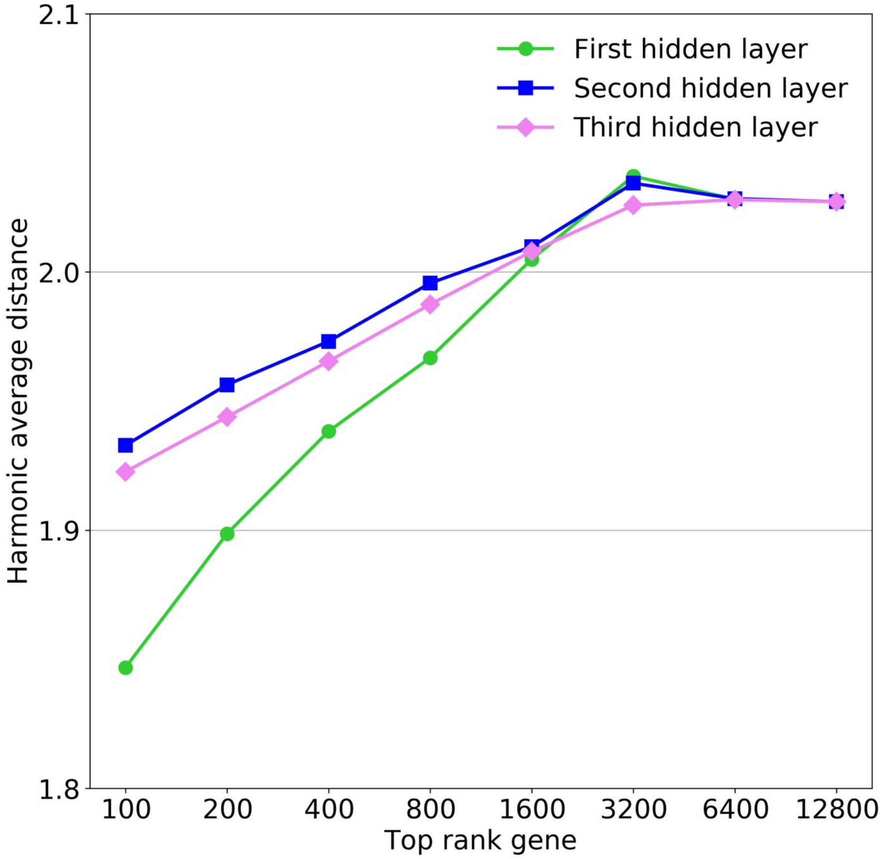

Supplementary Figure 4: demonstrates the harmonic average distance behavior of the top ranked genes on the basis of the first (green), second (blue) and third (violet) hidden layers of deep auto-encoder (deepAE) in the disease gene network. The deepAE was trained on the microarray data set. The disease gene network was constructed such that genes associated to the same disease were connected using BioSNAP[3]. Interstingly, the average distances of this network was signficantly correlated with the physical STRING PPI-network (Pearson correlation = 0.30 with parametric correlation test $P < 2.20 \times 10^{-16}$), and the association to this network was mostly in layer one, but also in the other layers.

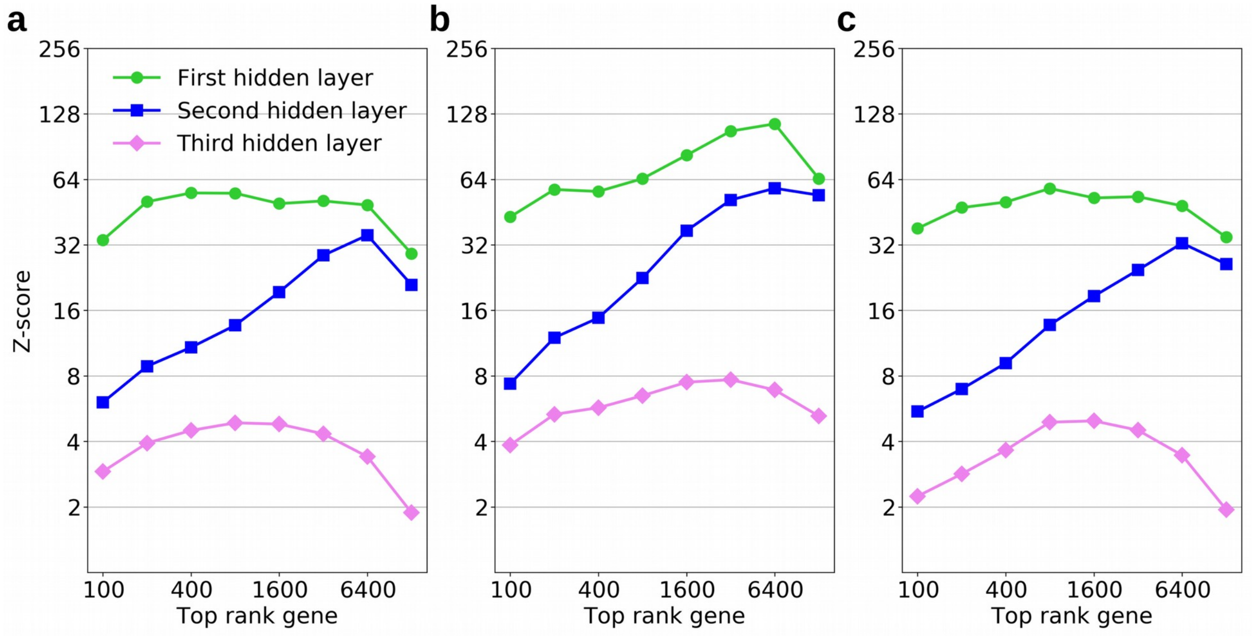

Supplementary Figure 5: (a) (b) and (c) demonstrate the coherence Z-score of the top ranked genes on the basis of the first (green), second (blue) and third (violet) hidden layers of the deep auto-encoder in Kegg, Reactome and GO-MF respectively[4]. Here, we define the coherence score of a given gene set as a maximum of -log10(p-value) across all the cluster annotations that have at least 10 genes in the overlap. The Z-score was computed with respect to the coherence score of the 1K random gene sets with similar sizes.

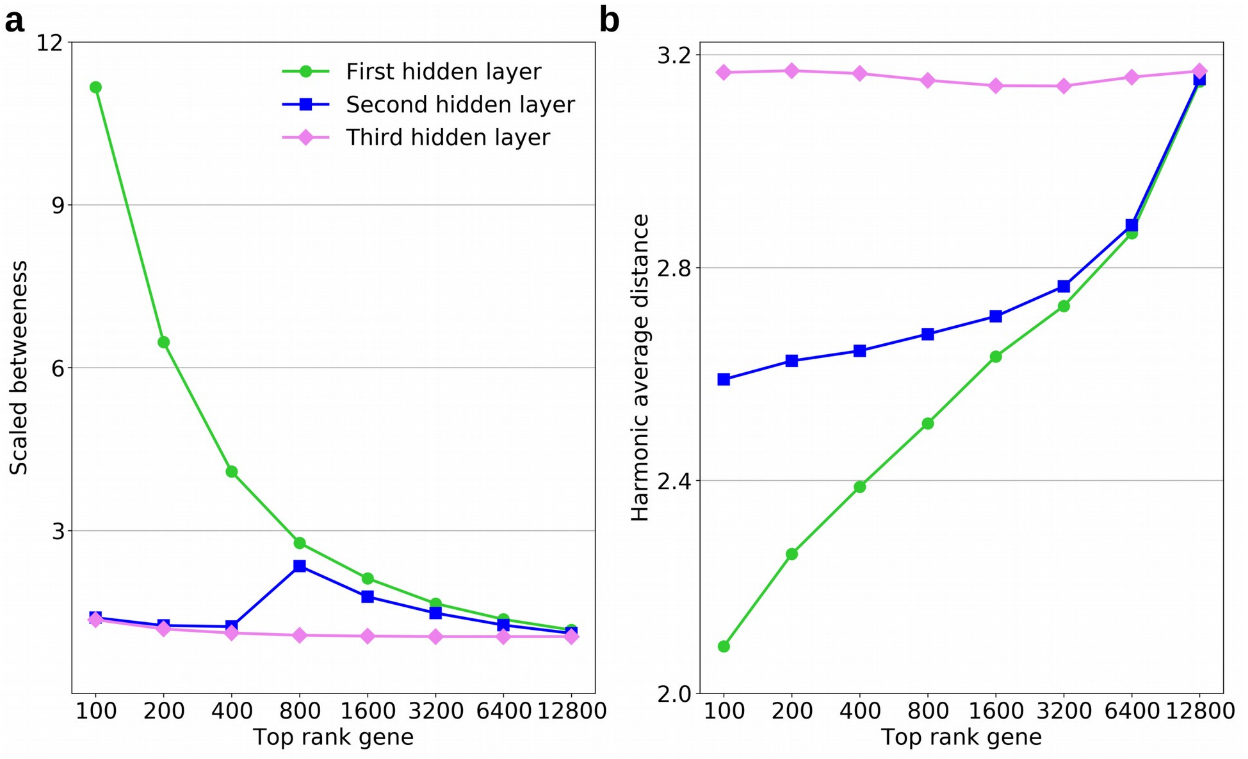

Supplementary Figure 6: Panel (a) demonstrate the betweenness centrality behavior of the top ranked genes on the basis of the first (green), second (blue) and third (violet) hidden layers of the denoised deep auto-encoder. Panel (b) shows the mean of harmonic average distances of the top rank genes based on each hidden node of the first, second and third hidden layers of the denoised deep auto-encoder respectively.

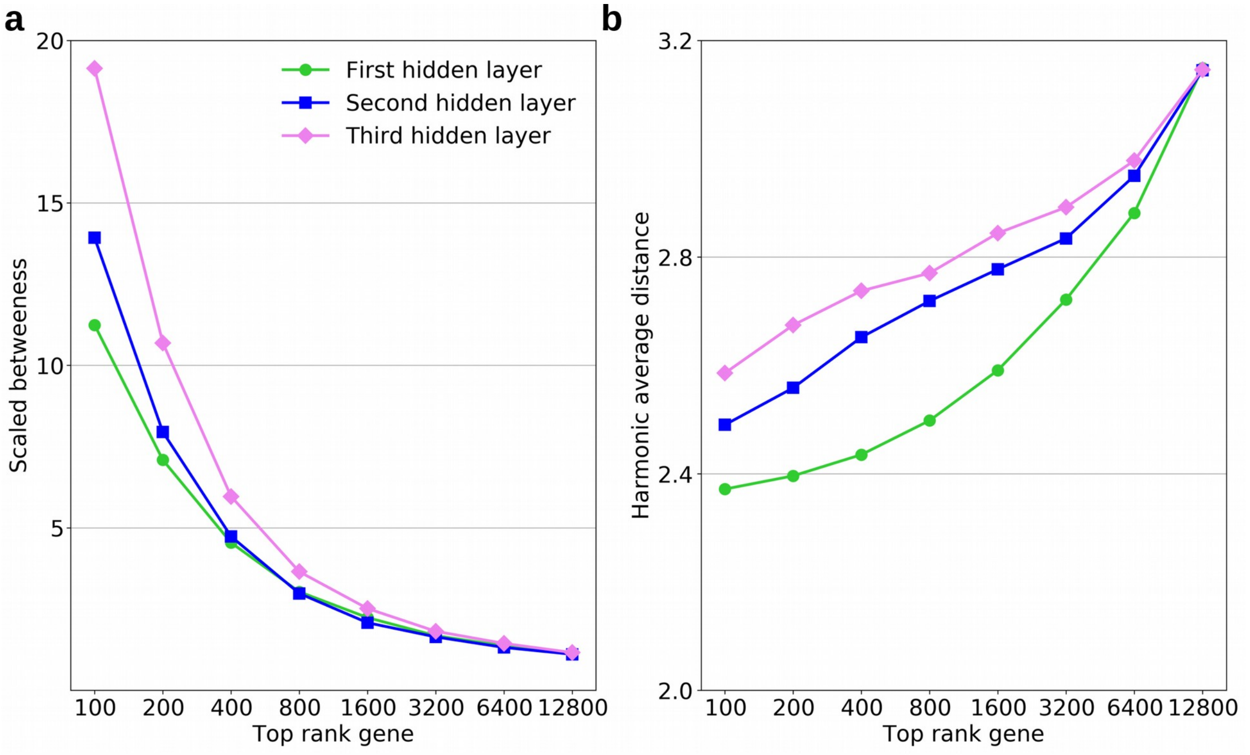

Supplementary Figure 7: Panel (a) demonstrate the betweenness centrality behavior of the top ranked genes on the basis of the first (green), second (blue) and third (violet) hidden layers of the sparsified deep auto-encoder trained on the microarray data set. Panel (b) shows the mean of harmonic average distances of the top rank genes based on each hidden node of the first, second and third hidden layers of the sparsified deep auto-encoder respectively.

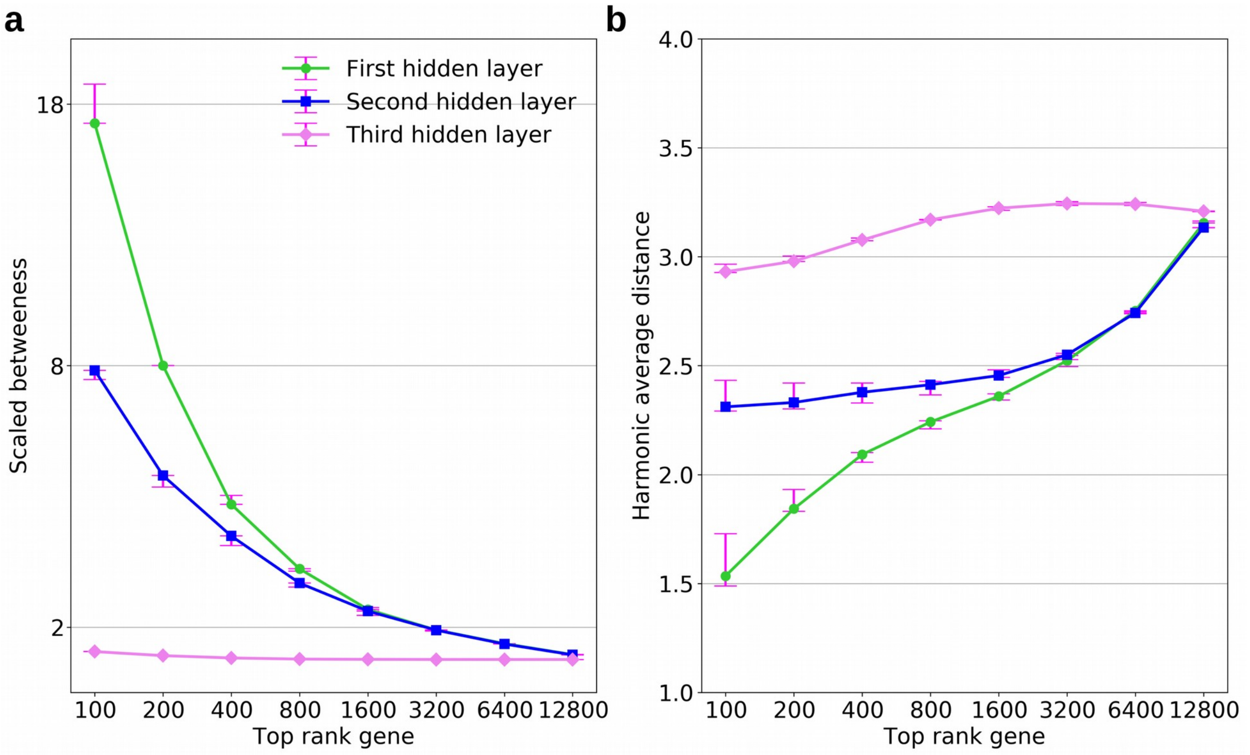

Supplementary Figure 8: Panel (a) demonstrate the betweenness centrality behavior of the top ranked genes on the basis of the first (green), second (blue) and third (violet) hidden layers of the deep auto-encoder trained on RNA-seq data with 20K randomly three times chosen samples (error bars are shown in magenta color) . Similarly, panel (b) shows the mean of harmonic average distances of the top rank genes based on each hidden node of the first, second and third hidden layers of the deep auto-encoder respectively.

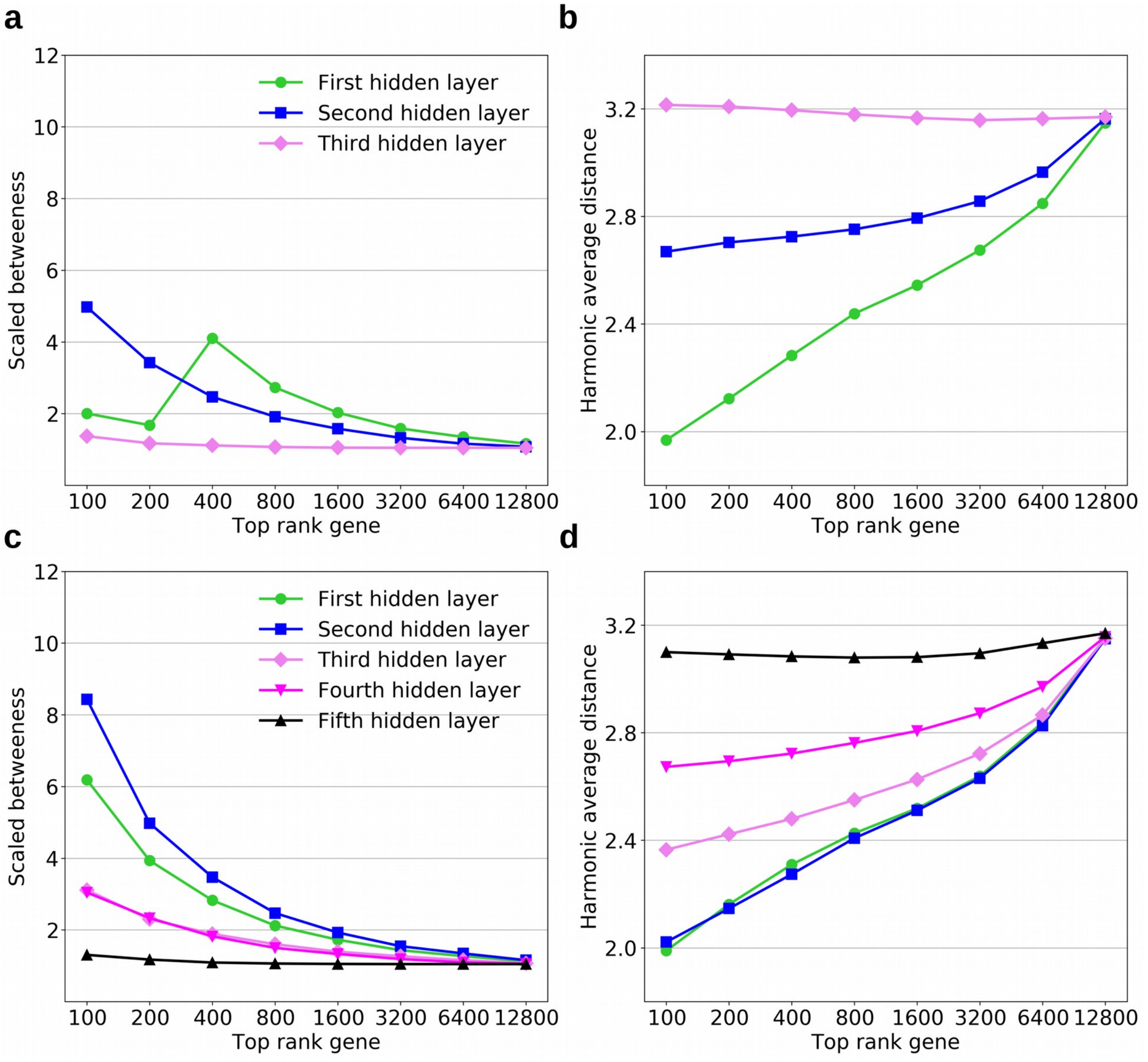

Supplementary Figure 9: Panel (a) demonstrate the betweenness centrality behavior of the top ranked genes on the basis of the first (green), second (blue) and third (violet) hidden layers of the 3 layer deep funnel shaped auto-encoder. Panel (b) shows the mean of harmonic average distances of the top rank genes based on each hidden node of the first, second and third hidden layers of the funnel shaped 3 layer deep auto-encoder trained on the microarray data set respectively. Similarly, Panel (c) shows the betweenness centrality behavior of the top ranked genes on the basis of the first (green), second (blue), third (violet), fourth (magenta) and fifth (black) hidden layers of the 5 layer deep funnel shaped auto-encoder. Panel (d) shows the mean of harmonic average distances of the top rank genes based on each hidden node of the first

(green), second (blue), third (violet), fourth (magenta) and fifth (black) hidden layers of the funnel shaped 5 layer deep auto-encoder respectively.

Supplementary References

1. Kibbe, W.A., Arze, C., Felix, V., Mitraka, E., Bolton, E., Fu, G., Mungall, C.J., Binder, J.X., Malone, J., Vasant, D. & Parkinson, H. Disease Ontology 2015 update: an expanded and updated database of human diseases for linking biomedical knowledge through disease data. *Nucleic Acids Res.* **43**, D545–D552 (2015).
2. Yu, G., Wang, L. G., Han, Y. & He, Q. Y. clusterProfiler: an R package for comparing biological themes among gene clusters. *OMICS* **16**, 284–287 (2012).
3. Zitnik, S. M. M. , Sosiĉ, R. & Leskovec J. BioSNAP Datasets: Stanford biomedical network dataset collection. http://snap.stanford.edu/biodata, (2018).
4. Gustafsson, M., Hörnquist, M. & Lombardi, A. Comparison and validation of community structures in complex networks. *Physica A*, **367**, 559-576 (2006).
